# PARP inhibition with rucaparib alone followed by combination with atezolizumab: Phase Ib COUPLET clinical study in advanced gynaecological and triple-negative breast cancers

**DOI:** 10.1038/s41416-024-02776-7

**Published:** 2024-07-06

**Authors:** Rebecca Kristeleit, Alexandra Leary, Ana Oaknin, Andres Redondo, Angela George, Stephen Chui, Aicha Seiller, Mario Liste-Hermoso, Jenna Willis, Colby S. Shemesh, Jim Xiao, Kevin K. Lin, Luciana Molinero, Yinghui Guan, Isabelle Ray-Coquard, Linda Mileshkin

**Affiliations:** 1 University College London Cancer Institute, London, UK; 2 School of Cancer and Pharmaceutical Sciences, King’s College London, London, UK; 3 Gustave Roussy, Villejuif, France; 4 Gynaecologic Cancer Programme, Vall d’Hebron Institute of Oncology (VHIO), Hospital Universitario Vall d’Hebron, Vall d’Hebron Barcelona Hospital Campus, Barcelona, Spain; 5 Medical Oncology Department, La Paz University Hospital-IdiPAZ, Madrid, Spain; 6 The Institute of Cancer Research, London, UK; 7 Royal Marsden NHS Foundation Trust, London, UK; 8 Product Development Oncology, Genentech Inc., South San Francisco, CA USA; 9 F. Hoffmann-La Roche Ltd, Basel, Switzerland; 10 Product Development Safety, Roche Products Ltd, Welwyn Garden City, UK; 11 Clinical Pharmacology Oncology, Genentech Inc, South San Francisco, CA USA; 12 Clovis Oncology, San Francisco, CA USA; 13 Translational Medicine, Genentech Inc., South San Francisco, CA USA; 14 Centre Leon Bérard, HESPER laboratory EA 7425, Université Claude Bernard Lyon Est, Lyon, France; 15 Department of Medical Oncology, Peter MacCallum Cancer Centre and University of Melbourne, Melbourne, VIC Australia; 16 Present Address: Guy’s and St Thomas’ NHS Foundation Trust and King’s College London, London, UK

**Keywords:** Ovarian cancer, Predictive markers, Targeted therapies, Cancer therapeutic resistance, Cancer immunotherapy

## Abstract

**Background:**

Combining PARP inhibitors (PARPis) with immune checkpoint inhibitors may improve clinical outcomes in selected cancers. We evaluated rucaparib and atezolizumab in advanced gynaecological or triple-negative breast cancer (TNBC).

**Methods:**

After identifying the recommended dose, patients with PARPi-naive BRCA-mutated or homologous recombination-deficient/loss-of-heterozygosity-high platinum-sensitive ovarian cancer or TNBC received rucaparib plus atezolizumab. Tumour biopsies were collected pre-treatment, during single-agent rucaparib run-in, and after starting combination therapy.

**Results:**

The most common adverse events with rucaparib 600 mg twice daily and atezolizumab 1200 mg on Day 1 every 3 weeks were gastrointestinal effects, fatigue, liver enzyme elevations, and anaemia. Responding patients typically had BRCA-mutated tumours and higher pre-treatment tumour levels of PD-L1 and CD8 + T cells. Markers of DNA damage repair decreased during rucaparib run-in and combination treatment in responders, but typically increased in non-responders. Apoptosis signature expression showed the reverse. CD8 + T-cell activity and STING pathway activation increased during rucaparib run-in, increasing further with atezolizumab.

**Conclusions:**

In this small study, rucaparib plus atezolizumab demonstrated acceptable safety and activity in BRCA-mutated tumours. Increasing anti-tumour immunity and inflammation might be a key mechanism of action for clinical benefit from the combination, potentially guiding more targeted development of such regimens.

**Clinical trial registration:**

ClinicalTrials.gov (NCT03101280).

## Background

The treatment landscape in gynaecological cancers and triple-negative breast cancer (TNBC) has changed substantially in recent years. Poly(ADP-ribose) polymerase (PARP) is a validated target for ovarian cancer and TNBC, especially for patients with homologous recombination deficiency (HRD), including loss-of-function mutations on *BRCA1*, *BRCA2*, and other genes important for DNA damage repair (DDR) and genomic loss of heterozygosity (gLOH). Following single- or double-strand DNA breaks, PARP is rapidly recruited to the damaged sites, signalling other DDR effector proteins to repair the damage. PARP inhibitors (PARPis) block the recruitment of effector proteins and DDR, and also (to varying degrees according to the agent) trap PARP at the DNA replication fork to block transcription and translation [1, 2]. In patients with HRD tumours, PARPis can induce synthetic lethality leading to decreased DDR, cell cycle arrest, and apoptosis [3]. PARPis have become part of standard therapy, initially as maintenance for platinum-sensitive recurrent ovarian cancer [4–6], and now in the front-line maintenance setting for ovarian cancer (with or without anti-angiogenic therapy) [7–9]. PARPis are also part of standard therapy in pre-treated metastatic *BRCA*-mutated breast cancer [10, 11] and as adjuvant therapy for *BRCA*-mutated early breast cancer [12].

Immunotherapy with immune checkpoint inhibitors (ICIs), including agents targeting programmed cell death-ligand 1 (PD-L1) or programmed cell death protein 1 (PD-1), has revolutionised cancer care for many solid tumours by potentially either reinvigorating exhausted tumour-specific cytotoxic CD8 + T cells or recruiting newly activated immune cells (ICs) to tumour sites [13]. This shifts the balance of the overall tumour immune microenvironment toward anti-tumour immune response. ICIs have become a standard of care in subsets of some tumour types, demonstrating clinically significant benefit in PD-L1-positive metastatic TNBC [14–17] and in early TNBC irrespective of PD-L1 status [18–20], but minimal efficacy in ovarian cancer [21–25].

As the treatment landscape evolves, clinical trials combining these approaches have been initiated, some of which include translational research objectives [26–28]. While such strategies have shown benefit in some patients, there remains a need to improve understanding of the underlying disease biology, mechanisms of action (MoAs), and the impact of these treatments within different subsets of patients, to try to determine whether precision medicine and individualised therapy can move into mainstream clinical practice in these settings.

Preclinical studies suggest that PARPis may synergise with ICIs to enhance anti-tumour immunity. First, reduced DDR resulting from PARPi leads to an accumulation of cytosolic DNA, which in turn activates the DNA-sensing cyclic GMP–AMP synthase (cGAS) stimulator of interferon genes (STING) pathway and increases type I interferons and other chemoattractant secretion [29]. Second, interferons and chemokines can attract ICs into the tumour vicinity and increase PD-L1 expression on tumour cells (TCs), thereby enhancing the effect of anti-PD-(L)1 agents [1]. PD-L1 expression on TCs can also be induced by an interferon-independent mechanism upon PARPi treatment [30]. Third, dead TCs can release tumour neoantigens and activate CD8 + T cells [1].

The predictive role of *BRCA* mutation/HRD for PARPi benefit in ovarian cancer has been observed consistently [31], as has the role of PD-L1 positivity for ICIs in metastatic TNBC [14, 16]. However, the biomarker interplay of *BRCA1/2* and PD-L1 expression in patients receiving PARPis and ICIs in combination has not been explored in the clinical setting, and is relatively poorly understood. Translational work suggests that patients with *BRCA*-mutated ovarian cancer, which have more protein-coding mutations that can potentially be targeted by the immune system, may show enhanced response to ICIs [32]. These tumours also have higher PD-1 and PD-L1 expression in intratumoral ICs, and an elevated number of CD3-positive and CD8-positive tumour-infiltrating lymphocytes (TILs) [32]. This led to the hypothesis that immunotherapy targeting PD-(L)1 may preferentially benefit patients with *BRCA*-mutated or HRD tumours, although this effect was not seen in post hoc analyses of the IMagyn050 trial [33].

Therefore, we explored the combination of rucaparib (a PARPi) and atezolizumab (a monoclonal antibody targeting anti-PD-L1) in biomarker-selected populations of patients with advanced gynaecological cancers or TNBC. The study was designed to evaluate the safety profile and potential pharmacokinetic (PK) interactions of the combination. Collection of baseline and on-treatment tumour tissue biopsies provided a unique opportunity to examine exploratory endpoints assessing the effect of rucaparib and atezolizumab on DDR, cell cycle, cell death, and the tumour immune microenvironment, as well as baseline and longitudinal biomarker effects.

## Methods

COUPLET (NCT03101280) was a multi-centre, two-part, open-label, non-randomised, Phase Ib study evaluating the combination of rucaparib and atezolizumab in patients with advanced gynaecological cancer or TNBC. Part 1 (dose finding) aimed to determine the recommended dose of rucaparib in combination with atezolizumab for further evaluation in Part 2 (dose expansion).

The primary objective was to evaluate the safety and tolerability of the combination by identifying dose-limiting toxicities (DLTs; Supplementary Table 1) that determined the maximum tolerated dose and identifying a potential recommended dose and schedule for subsequent assessments. Safety endpoints included the incidence, nature, and severity of adverse events (AEs) and DLTs according to National Cancer Institute Common Terminology Criteria for Adverse Events version 4.03. Secondary efficacy endpoints comprised objective response rate (ORR) according to Response Evaluation Criteria in Solid Tumours (RECIST) version 1.1, duration of response, progression-free survival (PFS) according to RECIST version 1.1, and overall survival (OS). The PK objective was to determine the PK of rucaparib and atezolizumab combination therapy in plasma and serum, with pre-specified parameters including steady-state maximum concentration observed (*C*_max_), time to maximum concentration (*T*_max_), and area under the concentration–time curve (AUC) for rucaparib and serum concentration of atezolizumab in Part 1, and minimum concentration during the dosing interval or trough concentration (*C*_min_) of rucaparib and serum concentration of atezolizumab in Part 2. The incidence of antidrug antibodies (ADAs) to atezolizumab was an additional secondary endpoint. The study also included pre-specified exploratory biomarker objectives: to evaluate predictive and pharmacodynamic biomarkers of response or resistance to combined rucaparib and atezolizumab therapy in tumour biopsies and blood; and to evaluate additional biomarkers of HRD and novel biomarkers and correlate them with response and/or clinical outcome, including efficacy and safety. Supplementary Table 2 describes biomarker-evaluable populations for the different assays. No formal statistical analyses were planned.

In Part 1 (dose-finding phase), patients with histologically confirmed ovarian or endometrial cancer who had received at least one prior line of therapy for metastatic disease received varying doses of oral rucaparib (400 mg twice daily in the first cohort, 600 mg twice daily in the second cohort, decreasing to 500 mg in the third cohort if necessary) in combination with a fixed dose of intravenous atezolizumab 1200 mg on Day 1. Cycles were repeated every 21 days and continued until unacceptable toxicity or disease progression if, in the investigator’s opinion, the patient would not derive clinical benefit from continued study treatment. It was planned to enroll ~six to 18 patients in up to three dose cohorts. The highest dose level demonstrating an acceptable safety profile in at least six patients, with fewer than one-third of patients experiencing a DLT during the first 21 days, was considered the recommended Phase II dose. In Part 2 (dose-expansion phase), the recommended Phase II dose was evaluated further for safety, PK, and preliminary activity in two tumour-specific expansion cohorts with an initially targeted enrollment of up to ~50 patients in total. In Cohort 1, eligible patients had platinum-sensitive high-grade serous or grade 3 endometrioid epithelial ovarian, fallopian tube, or primary peritoneal cancer with tumours harbouring a deleterious germline or somatic *BRCA* mutation (t*BRCA*_mut_, Arm A) or a t*BRCA*-like molecular signature (tBRCA-wild type [_wt_]/loss of heterozygosity [LOH]_high_, Arm B). Patients had received one to two prior platinum-containing regimens, with progression >6 months after the last dose of the most recent platinum therapy. In Cohort 2 (Arm C), patients had t*BRCA*_mut_ or t*BRCA*_wt_/LOH_high_ TNBC and had received at least one prior line of chemotherapy (without cancer immunotherapy) in the metastatic setting. Part 2 treatment comprised a 21-day run-in period of single-agent rucaparib at the identified recommended rucaparib starting dose (run-in), followed by rucaparib in combination with intravenous atezolizumab 1200 mg on Day 1 (combination). Cycles were repeated every 21 days.

All patients (Parts 1 and 2) were required to have sufficient archival formalin-fixed paraffin-embedded (FFPE) tumour tissue available for planned analyses. In Part 2, biopsies were mandatory before starting rucaparib (if archival pre-treatment tissue was collected >90 days before treatment), after starting the rucaparib run-in phase before combination therapy, and at Days 15–21 of cycle 1 after initiating combination therapy. A further biopsy at disease progression was optional. Tumour and biomarker characteristics are described according to central review result. However, patients could be enrolled or assigned to a cohort based on local test results, to avoid delaying treatment initiation. In some cases, this resulted in a patient apparently being ineligible for the cohort in which they were treated. Additional inclusion criteria (beyond the disease-specific criteria for each cohort and arm mentioned above) included female aged ≥18 years, Eastern Cooperative Oncology Group performance status 0/1, life expectancy ≥3 months, measurable disease according to RECIST version 1.1, and adequate organ function. Patients were ineligible if they had previously received treatment with a PARPi, CD137 agonist, or other ICI (including but not limited to anti-PD-1, anti-PD-L1, and anti-CTLA-4 therapeutic antibodies).

After initiating study treatment, all AEs were to be reported until 30 days after the last dose of study treatment (90 days for serious AEs and AEs of special interest) or until the start of another anti-cancer therapy, whichever occurred earlier. Serious AEs and AEs of special interest that were considered related to study treatment were to be reported indefinitely. During the treatment period, tumours were assessed every 9 weeks during the first year and subsequently every 12 weeks until the end-of-treatment visit or radiographic disease progression based on RECIST version 1.1, as assessed by the investigator. For patients discontinuing study treatment for a reason other than disease progression or death, subsequent tumour assessments were to be performed every 12 weeks until radiographic disease progression based on RECIST version 1.1, initiation of further systemic cancer therapy, or death, whichever occurred first. Patients were asked to return 120 days after the last dose of atezolizumab for collection of blood samples for PK and ADA analyses. Non-compartmental analysis of PK data was performed when intensive collection was available.

### PD-L1 and CD8 immunohistochemistry

Immunohistochemistry (IHC) assays were performed at CellCarta (Antwerp, Belgium) on 4-µm FFPE tissue sections. PD-L1 protein level was assessed with the PD-L1 IHC SP142 assay (Ventana Medical Systems, Tucson, AZ, USA) [34] and was characterised according to PD-L1 expression on TCs (percentage of total TCs expressing PD-L1) and tumour-infiltrating ICs (proportion of tumour area occupied by PD-L1 staining ICs of any intensity). PanCK–CD8 duplex IHC used the SP239 anti-CD8 clone (catalogue no. M5394; Spring Bioscience, Pleasanton, CA) and the AE1/AE3/PCK26 anti-Pan-Keratin clone (catalogue no. 760-2135; Roche Tissue Diagnostics, Rotkreuz, Switzerland) and was scored using the manufacturer’s scoring methods (CD8+PanCK multiplex IHC assay; CellCarta, Gosselies, Belgium). The intratumoural CD8 + T-cell density was determined as the percentage of CD8-stained cells occupying the defined tumour area.

A multiplex CD3/FOXP3 immunofluorescence assay was performed to assess percentages of T-regulatory cells (Tregs) among all T cells (percentage of CD3/FOXP3 double-positive staining cells of all CD3-positive cells in the defined tumour area [35]).

### Genomic next-generation sequencing FoundationOne Panel

The FoundationOne (F1CDx) assay (FoundationOne, Cambridge, MA, USA) was performed retrospectively on FFPE tumour tissue section DNA samples from Part 1 patients. In Part 2, F1CDx assay results were used to pre-select patients with *BRCA* mutations and/or gLOH ≥16% (the refined cut-off established from ARIEL2 [36] and used in ARIEL3 [6]) for enrolment. The manufacturer’s algorithms were used for detailed content and mutation, gLOH, microsatellite instability (MSI), and tumour mutational burden (TMB) calling [37]. The gLOH algorithm has been validated only in tissue samples with >35% tumour content from ovarian cancer [6]. TMB and MSI were determined as previously described [38].

### RNA analyses

Macrodissection was performed on sections of resected or core needle biopsy FFPE tumour tissue to enrich tumour percentage to >50% before RNA extraction. RNA was extracted from four to five sections of 4-µm FFPE tissue from pre-treatment and on-study tissue resections and biopsies from patients enrolled in Part 2. RNA sequencing was performed using TruSeq RNA Exome technology (Illumina, San Diego, CA, USA) at Q2 Solutions (Durham, NC, USA). RNA sequencing reads were first aligned to ribosomal RNA sequences to remove ribosomal reads. The remaining reads were aligned to the human reference genome (National Center for Biotechnology Information Build 38) using Genomic Short-read Nucleotide Alignment Program (GSNAP) version 2013-11-01. To quantify gene expression levels, the number of reads mapped to the exons of each RefSeq gene was calculated using the functionality provided by the R/Bioconductor package GenomicAlignments. For each cohort, raw counts for genes were trimmed mean of M-values (TMM)-normalised based on size factors as calculated with CalcNormFactors in the edgeR package. The TMM-normalised counts were subsequently voom transformed with the voom function in the limma package, resulting in normalised log2 counts per million (CPM) data. Gene features were filtered for low expression (defined as median CPM above the 25th percentile) and for low variance (defined as median CPM coefficient of variation above the 25th percentile). For RNA sequencing data, patient-matched paired *t* tests were performed to identify differentially expressed genes (DEGs) comparing run-in vs. pre-treatment, post-combination vs. run-in, and post-combination vs. pre-treatment, regardless of response status. Significant DEGs (*P* < 0.05 and >twofold change) were then analysed for their pathway enrichment through Ingenuity Pathway Analysis (IPA; Qiagen, Redwood City, CA, USA). Gene signatures are described in Supplementary Table 3.

The study was approved by the Institutional Review Board or Ethics Committee at each site before study initiation (NHS Health Research Authority reference 16/LO/2106). All patients provided written informed consent before undergoing any study-specific procedures. The study was conducted in full conformance with the International Conference on Harmonisation E6 guidelines for Good Clinical Practice and the principles of the Declaration of Helsinki, or the laws and regulations of each country if they afforded greater protection to the individual.

## Results

### Patient population

Patients were enrolled from eight centres in the UK, France, Australia, and Spain between May 4, 2017, and February 18, 2019. The trial was discontinued prematurely because of slower than anticipated recruitment, after enrolment of nine patients in Part 1 (dose finding) and 19 patients in Part 2 (dose expansion) (10 patients with t*BRCA*_mut_ ovarian cancer in Arm A, four patients with t*BRCA*_*wt*_/LOH_high_ ovarian cancer in Arm B, and five patients with t*BRCA*_mut_ or LOH_high_ TNBC in Arm C).

Baseline characteristics are shown in Table 1. In Part 1, seven patients had ovarian-type cancer, and two had endometrial cancer.

**Table 1:** Baseline characteristics (based on local assessment).

Characteristic	Part 1 (dose finding)	Part 2 (dose expansion)	
	Cohorts 1 and 2 (*n* = 9)	Ovarian cancer, Arms A + B (*n* = 14)	TNBC, Arm C (*n* = 5)
Median age, years (range)	61 (48–77)	60 (34–80)	49 (30–57)
Region, *n* (%)			
Europe	6 (67)	13 (93)	5 (100)
Australia	3 (33)	1 (7)	0
Race, *n* (%)			
White	9 (100)	14 (100)	1 (20)
Black or African American	0	0	1 (20)
Unknown	0	0	3 (60)
Baseline ECOG PS, *n* (%)			
0	7 (78)	11 (79)	3 (60)
1	2 (22)	3 (21)	2 (40)
*BRCA1* or *BRCA2* mutation status, *n* (%)			
Deleterious variant detected	2 (22)	9 (64)	4 (80)
Deleterious variant not detected	7 (78)	1 (7)	0
Missing	0	4 (29)	1 (20)
LOH status, *n* (%)			
≥16%	2 (22)	9 (64)	4 (80)
<16%	0	0	0
Missing/not done	7 (78)	5 (36)	1 (20)
PD-L1 status, *n* (%)			
IC < 1% (negative)	5 (56)	4 (29)	1 (20)
IC ≥ 1% (positive)	4 (44)	7 (50)	0
Missing/not done	0	3 (21)	4 (80)

*ECOG PS* Eastern Cooperative Oncology Group performance status, *IC* immune cell, *LOH* loss of heterozygosity, *PD-L1* programmed cell death-ligand 1, *TNBC* triple-negative breast cancer.

### Treatment exposure

The data cut-off for the final analysis reported here was August 5, 2020 (last patient last visit). By this date, all patients had discontinued from treatment and the study. Disease progression was mentioned as a reason for discontinuation of both atezolizumab and rucaparib in all but five patients. The last enrolled patient with TNBC was still deriving clinical benefit at the data cut-off and was transitioned to another trial enabling continued combination treatment with rucaparib plus atezolizumab. The median duration of follow-up was 15.2 months in Part 1 (range 5.0–36.1 months), 17.2 months in Part 2 Cohort 1 (ovarian cancer; range 2.9–27.8 months) and 10.7 months in Part 2 Cohort 2 (TNBC; range 5.8–19.1 months). Treatment exposure is shown in Supplementary Table 4.

### Safety

Safety is summarised in Table 2. There were no DLTs in Part 1 (dose-finding phase evaluating rucaparib 400 or 600 mg twice daily) and the recommended dose for Phase II evaluation was rucaparib 600 mg twice daily in combination with atezolizumab 1200 mg on Day 1, repeated every 21 days. Across the entire study, there were no fatal AEs. Three patients discontinued study treatment because of AEs. The most common AEs across both parts of the study were gastrointestinal (nausea, diarrhoea, abdominal pain, constipation), fatigue, liver enzyme elevations, and anaemia (Table 3). The only grade 3/4 AEs of special interest for atezolizumab were grade 3 increased transaminase levels (in one patient receiving rucaparib 600 mg in Part 1 and three patients in Part 2 [one in each arm]) and maculopapular rash (in one patient receiving rucaparib 400 mg in Part 1 and the patient at 600 mg mentioned above with increased transaminase levels). Most cases of increased transaminase levels resolved (after rucaparib dose reduction in three patients). There were no cases of myelodysplastic syndrome or acute myeloid leukaemia. One patient experienced grade 4 bone marrow failure, which was considered symptomatic of disease progression but also related to rucaparib and concurrent illness.

**Table 2:** Overview of safety.

Patients, *n* (%)	Part 1 (dose finding)		Part 2 (dose expansion)			
	Cohort 1 (*n* = 3)	Cohort 2 (*n* = 6)	Ovarian cancer, Arms A + B (*n* = 14)		TNBC, Arm C (*n* = 5)	
			Run-in	Post run-in	Run-in	Post run-in
Any grade AE	3 (100)	6 (100)	12 (86)	14 (100)	5 (100)	5 (100)
Grade 3/4	1 (33)	4 (67)	4 (29)	10 (71)	2 (40)	1 (20)
Grade 5	0	0	0	0	0	0
Treatment-related AE	3 (100)	6 (100)	12 (86)	12 (86)	4 (80)	3 (60)
AE leading to treatment discontinuation	0	1 (17)^a^	0	2 (14)^b^	0	0
AE leading to treatment modification/interruption	3 (100)	4 (67)	3 (21)	8 (57)	1 (20)	1 (20)
Atezolizumab	1 (33)	1 (17)	0	5 (36)	0	1 (20)
Rucaparib	3 (100)	4 (67)	3 (21)	8 (57)	1 (20)	1 (20)

*AE* adverse event, *TNBC* triple-negative breast cancer.

^a^Atezolizumab was discontinued because of non-serious grade 1/2 diarrhoea starting on Day 72.

^b^Arm A, 1 patient discontinued rucaparib because of persistent grade 2/3 neutropenia starting on Day 253 and atezolizumab because of grade 2 pericarditis starting on Day 310; 1 patient discontinued atezolizumab and rucaparib because of serious grade 3 acute kidney injury and grade 4 bone marrow failure with non-serious grade 2/3 anaemia and grade 1–3 decreased platelet count, all starting on Day 63.

**Table 3:** Most common adverse events (any grade in ≥50%; grade ≥3 in ≥25%).

Patients with adverse event, *n* (%)	Part 1 (dose finding)				Part 2 (dose expansion)					
	Cohort 1 (rucaparib 400 mg) (*n* = 3)		Cohort 2 (rucaparib 600 mg) (*n* = 6)		Ovarian cancer				TNBC	
					Arm A (t*BRCA*_mut_) (*n* = 10)		Arm B (t*BRCA*_wt_/LOH_high_) (*n* = 4)		Arm C (*n* = 5)	
	Any grade	Grade ≥ 3	Any grade	Grade ≥ 3	Any grade	Grade ≥ 3	Any grade	Grade ≥ 3	Any grade	Grade ≥ 3
Nausea	2 (67)	0	5 (83)	0	7 (70)	0	4 (100)	0	3 (60)	0
Fatigue	2 (67)	0	6 (100)	1 (17)	6 (60)	0	1 (25)	0	0	0
Diarrhoea	2 (67)	0	6 (100)	0	3 (30)	0	0	0	2 (40)	0
ALT increased	0	0	3 (50)	0	3 (30)	1 (10)	3 (75)	1 (25)	3 (60)	1 (20)
AST increased	0	0	3 (50)	0	3 (30)	1 (10)	2 (50)	1 (25)	3 (60)	0
Anaemia	0	0	3 (50)	0	4 (40)	3 (30)	2 (50)	1 (25)	1 (20)	1 (20)
Abdominal pain	3 (100)	0	2 (33)	0	3 (30)	1 (10)	1 (25)	0	1 (20)	0
Constipation	2 (67)	0	3 (50)	0	2 (20)	0	0	0	3 (60)	0
Pruritus	2 (67)	0	3 (50)	0	3 (30)	0	1 (25)	0	0	0
Decreased appetite	0	0	3 (50)	0	3 (30)	1 (10)	1 (25)	0	1 (20)	0
Headache	1 (33)	0	3 (50)	0	1 (10)	0	0	0	1 (20)	0
Neutropenia	0	0	1 (17)	1 (17)	3 (30)	3 (30)	1 (25)	1 (25)	0	0
Thrombocytopenia	0	0	1 (17)	1 (17)	1 (10)	1 (10)	2 (50)	0	1 (20)	0
Dysphagia	0	0	3 (50)	0	2 (20)	0	0	0	0	0
Rash maculopapular	2 (67)	1 (33)	1 (17)	1 (17)	0	0	0	0	0	0
Back pain	1 (33)	0	1 (17)	0	0	0	2 (50)	0	2 (40)	0
Dysgeusia	0	0	1 (17)	0	0	0	2 (50)	0	0	0
Neutropenic sepsis	0	0	0	0	1 (10)	1 (10)	1 (25)	1 (25)	0	0
Pancytopenia	0	0	0	0	0	0	1 (25)	1 (25)	0	0

*ALT* alanine aminotransferase, *AST* aspartate aminotransferase, *LOH* loss of heterozygosity, *mut* mutated, *tBRCA* tumour *BRCA* gene, *wt* wild type.

### Pharmacokinetics

Rucaparib PK analysis showed relatively rapid absorption and dose-proportional kinetics between 400 and 600 mg (Table 4). Rucaparib *T*_max_ ranged from 1.5 to 6 h. There was moderate-to-high inter-individual variability in exposure ranging from 27.4–46.8% for *C*_max_, 28.6–88.7% for *C*_min_, and 21.2–41.6% for AUC across Parts 1 and 2. Steady-state profiles were relatively flat, and *C*_max_, AUC, and *C*_min_ were highly correlated. Sparse PK sampling of atezolizumab showed similar atezolizumab exposure regardless of the rucaparib dose. There was low-to-moderate inter-patient variability, ranging from 7.0–35.3% for *C*_max_ and 17.9–32.0% for *C*_min_, and all trough concentrations (*C*_trough_) exceeded the target concentration for maximal receptor occupancy of 6 μg/mL. Among 28 patients evaluable for ADA, one (4%) was positive for treatment-emergent ADAs.

**Table 4:** Summary of Cycle 1 PK analyses.

Parameter	Part 1 (dose finding)		Part 2 (dose expansion)		
	Cohort 1	Cohort 2	Ovarian cancer		TNBC, Arm C
			Arm A (t*BRCA*_mut_)	Arm B (t*BRCA*_wt_/LOH_high_)	
**Atezolizumab Cycle 1 arithmetic mean (CV)**	**Sparse PK sampling**		**Sparse PK sampling**		
*C*_max_, μg/mL	(*n* = 3) 429 (7.0)	(*n* = 6) 500 (19.4)	(*n* = 10) 469 (18.8)	(*n* = 4) 438 (9.4)	(*n* = 4) 425 (35.3)
*C*_min_, μg/mL	(*n* = 3) 84.3 (32.0)	(*n* = 5) 96.0 (23.8)	(*n* = 10) 77.2 (22.1)	(*n* = 4) 95.7 (17.9)	(*n* = 4) 98.7 (26.3)
**Rucaparib arithmetic mean (CV)**	**Intensive PK sampling**		**Sparse PK sampling after 21-day run-in**		
*C*_max_, ng/mL	(*n* = 2) 2270 (27.4)	(*n* = 6) 3185 (46.8)	–	–	_
*C*_min_, ng/mL	(*n* = 2) 1730 (28.6)	(*n* = 6) 2650 (28.9)	(*n* = 9) 1910 (88.7)	(*n* = 4) 1500 (75.7)	(*n* = 5) 1180 (52.5)
AUC_last_ (h·ng/mL)	(*n* = 2) 15 072 (21.2)	(*n* = 6) 22 082 (41.6)	–	–	–

*AUC*_*last*_ area under the concentration–time curve to the last measurable plasma concentration, C_*max*_ maximum concentration observed, C_*min*_ minimum concentration during the dosing interval or trough concentration, *CV* coefficient of variation, *LOH* loss of heterozygosity, *mut* mutated, *PK* pharmacokinetic, *tBRCA* tumour *BRCA* gene, *TNBC* triple-negative breast cancer, *wt* wild type.

### Efficacy

**Fig. 1:**
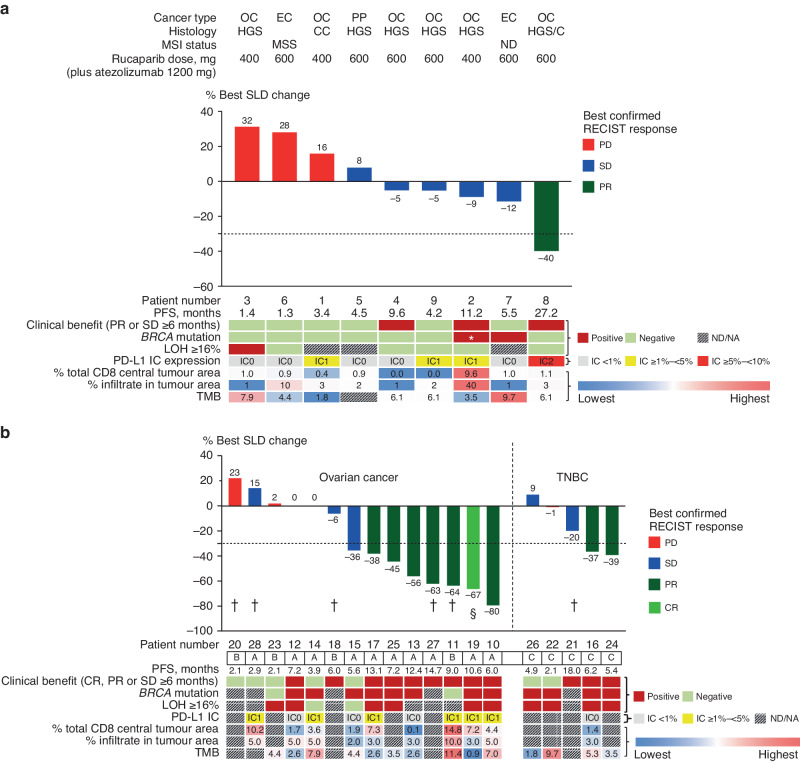
Baseline genetic and tumour immune microenvironment characteristics. **a** Part 1 (all-comer ovarian and endometrial cancer dose-finding phase). **b** Part 2 (dose expansion). Patients in panels a and b are sorted from left to right by greatest reduction in SLD of target lesions. Tumour and biomarker characteristics are described according to the result from central review. However, patients could be enrolled or assigned to a cohort on the basis of local test results, to avoid delays in treatment initiation. In some cases, this resulted in a patient apparently being ineligible for the cohort in which they were treated. ^*^BRCA1 mutation (p.D120H) with unknown functional consequence. ^†^Patients who had NA/ND status for both *BRCA* mutation and LOH were enrolled based on local testing results. ^§^Patient achieved CR despite best SLD change of –67% because the primary lesion (lymph node) decreased to <10 mm. CC clear-cell, CR complete response, EC endometrial cancer, HGS high-grade serous, HGS/C high-grade serous/carcinosarcoma, IC immune cell, LOH loss of heterozygosity, MSI, microsatellite instability, MSS microsatellite stable, NA not available, ND not done, OC ovarian cancer, PD progressive disease, PD-L1 programmed cell death-ligand 1, PFS progression-free survival, PP primary peritoneal cancer, PR partial response, RECIST Response Evaluation Criteria in Solid Tumours, SD stable disease, SLD sum of longest diameters, TMB tumour mutational burden, TNBC triple-negative breast cancer.

In Part 1, one patient with t*BRCA*_wt_/LOH_low_ high-grade serous ovarian cancer/carcinosarcoma treated with rucaparib 600 mg achieved a partial response (PR) (17% response rate, 95% confidence interval [CI]: 0–64%) and five achieved stable disease (SD). In two patients with SD (both with high-grade serous ovarian cancer), the disease was stabilised for ≥6 months. Meaningful interpretation of biomarker findings is limited by the small patient numbers (Fig. 1a). However, of two patients with t*BRCA1*_mut_ ovarian cancer, one showed clinical benefit (SD and PFS of 11.2 months). The only responder in Part 1 had a duration of response of 25.9 months and PFS of 27.2 months, and the highest PD-L1 IC expression (7%) among all nine enrolled patients. The patient achieving SD with PFS of 11.2 months had PD-L1 expression of 2% in ICs and the highest percentage of IC infiltrates (40%) and CD8 + T cells (10%) among all patients in Part 1. Of note, none of the nine patients enrolled in Part 1 had detectable PD-L1 expression in TCs (TC data not shown), TMB ≥ 10 mutations/megabase (Mut/Mb), or MSI high.

In Part 2, objective responses were achieved in six of 10 patients in Arm A (t*BRCA*_mut_ ovarian cancer by local testing; 60% response rate, 95% CI: 26–89%, including one complete response [CR]) and one of four patients in Arm B (t*BRCA*_wt_/LOH_high_ ovarian cancer by local testing; 25% response rate, 95% CI: 1–81%). Additionally, one patient with SD in Arm A and one in Arm B had PFS of ≥6 months. Among the five patients in Part 2 Arm C (t*BRCA*_mut_ or LOH_high_ TNBC according to local testing), two achieved a PR (40% response rate, 95% CI: 5–85%) and one patient with SD had PFS of 18.0 months.

Four of the five patients with ovarian cancer whose tumours were PD-L1 IC ≥ 1% and high CD8 (≥ median [4%]) achieved a PR or CR (Fig. 1b). One responder with ovarian cancer had TMB ≥ 10 Mut/Mb and t*BRCA*_wt_/LOH_unknown_ (central assessment). None of the Part 2 patients had detectable PD-L1 expression on TCs or MSI high. The longest PFS among patients with ovarian cancer was 14.7 months (duration of response 12.9 months, OS censored at 17.8 months). According to local assessment, this patient had t*BRCA*_mut_ disease.

Overall, 19 patients (68%) across all treatment arms had died by the analysis cut-off date. Death was from disease progression in 15 patients and unknown causes in four patients (Supplementary Table 5).

### Longitudinal biomarker analyses

**Fig. 2:**
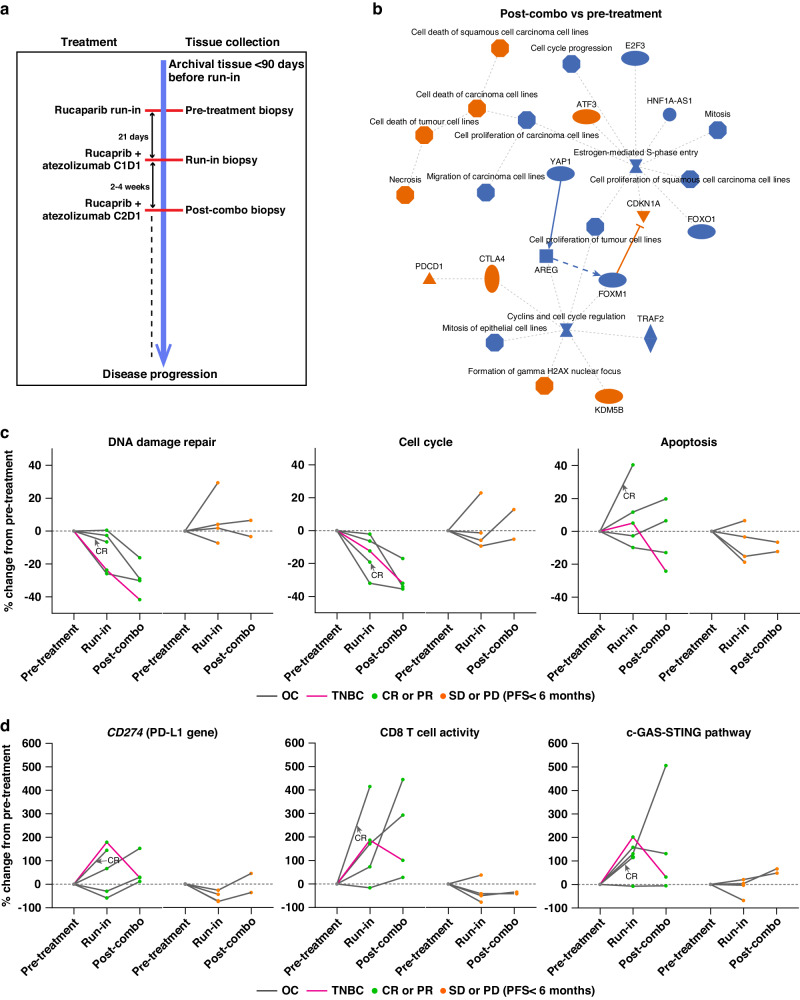
Dynamic changes of tumour intrinsic and tumour immune microenvironment molecular features in longitudinal biopsies. RNA sequencing transcriptome profiling was done in mandatory tumour biopsy samples collected before treatment, during rucaparib run-in (run-in), and at C2D1 of rucaparib plus atezolizumab (post-combination). **a** Schematic of biopsy collection timepoints in relation to treatment schedule. **b** Summary of enriched pathways (Ingenuity pathway analysis) from significantly differentially expressed genes (≥twofold change, *P* < 0.05) in paired post-combination vs. pre-treatment biopsies. Orange symbols: upregulated gene pathways; blue symbols: downregulated gene pathways. **c** Dynamic gene expression levels in the PARPi-regulated pathways for DDR, cell cycle, and apoptosis pathways. **d** Dynamic expression changes in immune signatures for CD274 (PD-L1 gene) and signatures representing CD8 T-cell activity and cGAS-STING. Each line plot in (**c**, **d**) represents a patient with confirmed CR or PR (left-hand panels) or SD or PD (right-hand panels). CnDn Cycle n Day n, cGAS cyclic GMP–AMP synthase, CR complete response, DDR DNA damage repair, OC ovarian cancer, PARPi poly(ADP-ribose) polymerase inhibitor, PD progressive disease, PD-L1 programmed cell death-ligand 1, PFS progression-free survival, PR partial response, SD stable disease, STING stimulator of interferon genes, TNBC triple-negative breast cancer.

Figure 2a and Supplemental Table 2 summarise baseline and longitudinal biomarker sampling timepoints, datasets, and assays. Changes in the biological pathways associated with rucaparib MoA were apparent after combination therapy, including decreased cell cycle/proliferation and increased cell death and apoptosis RNA-based signatures (Fig. 2b). After the rucaparib run-in, DDR decreased in four of five responders but only one of four non-responders (by <10%; Fig. 2c). After combination therapy, DDR decreased further from run-in in all responders but in only one of two non-responders. The decrease in cell cycle signature expression post-combination vs. run-in was consistent with the trend in DDR and was seen only in responders. Conversely, three of the four non-responders had an increase after run-in or post-combination (Fig. 2c), indicating that tumours continued to grow and were resistant to therapy. In contrast, apoptosis signature expression increased in three of five responding patients during run-in, and increased further after combination therapy in two patients, whereas only one of four patients with SD or progressive disease (PD) had a modest (< 10%) increase in apoptosis.

Although global DEGs and IPA exploring the dynamics of immune modulation did not reveal any significantly enriched pathways related to tumour immune microenvironment (Fig. 2b), analysis of the dynamics of curated immune genes and signatures (Supplementary Table 3) revealed increased *CD274* expression after rucaparib run-in in three of five responders but not in non-responders (Fig. 2d). However, after the addition of atezolizumab, both responders and non-responders had increased *CD274* levels compared with run-in. During rucaparib run-in, there were dramatic increases in CD8 + T-cell activity (>50% increase) and cGAS-STING pathway activation (>100% increase) in four of five responders, and sustained or further increases after adding atezolizumab in two responders. In contrast, none of the four patients with SD/PD had >50% change in T-cell activity or cGAS-STING pathway at run-in and only one patient with SD had an increase (73%) in cGAS-STING pathway expression after combination therapy (Fig. 2d).

**Fig. 3:**
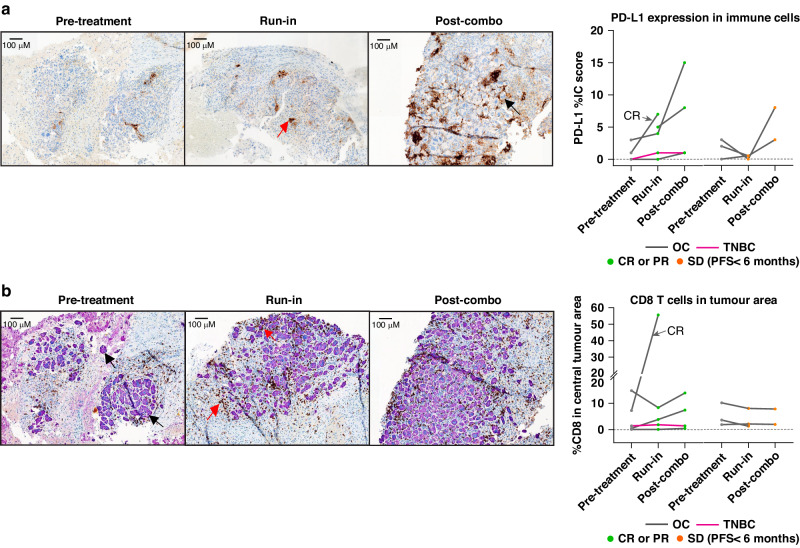
Dynamic changes in PD-L1 protein expression in ICs and CD8 T-cell infiltration in longitudinal biopsies. **a** Left: representative PD-L1 IHC images in patient 10 (PR) at pre-treatment, run-in, and post-combination; Right: line plot graph of PD-L1 staining in ICs at different timepoints in responders (CR or PR) and non-responders (SD or PD). PanCK-positive tumour cells are purple and CD8-positive cells are brown. **b** Left: representative duplex IHC images for PanCK (tumour epithelial cells, magenta) and CD8 (T cells, brown) in patient 10 at different timepoints; Right: line plot graph summarising the percentage of CD8 infiltration over time by clinical outcomes and tumour indication. The black arrows point to PanCK (purple) stained tumour cells and the red arrows point to stained CD8 cells (brown). CR, complete response, IC immune cell, IHC immunohistochemistry, OC ovarian cancer, PD progressive disease, PD-L1 programmed cell death-ligand 1, PR partial response, SD stable disease, TNBC triple-negative breast cancer.

Increased expression of PD-L1 protein and CD8 + T-cell infiltration post run-in and combination therapy were validated with IHC analysis (Fig. 3).

We found no clear trend in Treg changes in association with RECIST response to treatment (Supplementary Fig. 1). In addition, among the eight patients with both baseline and longitudinal biopsies, changes in tumour-infiltrating CD8 + T cells generally (seven of eight patients) tracked with those of PD-L1 ICs (Supplemental Fig. 2), but Treg changes were more variable.

## Discussion

In study Part 1 (dose-escalation phase), there were no DLTs and the recommended dose for Phase II evaluation was rucaparib 600 mg twice daily in combination with atezolizumab 1200 mg on Day 1, repeated every 21 days. This regimen demonstrated an acceptable safety profile consistent with the known effects of rucaparib and atezolizumab. There were no new safety signals and treatment discontinuation was infrequent. The PK profiles for both drugs were as expected. However, early study discontinuation due to poor enrollment in the dose-expansion phase limits conclusions on safety and efficacy.

Responses to rucaparib and atezolizumab combination therapy were observed predominantly in patients with *BRCA*-mutated tumours. These patients derived long-term benefit from therapy, consistent with published findings characterising long-term responders in two prospective studies of rucaparib in ovarian cancer [39]. The Phase II MEDIOLA study of durvalumab plus olaparib in PARPi-naive patients with germline *BRCA1/2*-mutant platinum-sensitive recurrent ovarian cancer showed a 72% ORR, median duration of response of 10.2 months, and median PFS of 11.1 months [40]. Although the 60% ORR with rucaparib and atezolizumab in *BRCA*-mutated ovarian cancer is numerically lower than the 72% ORR with durvalumab plus olaparib [40] and the 72% ORR with olaparib alone in patients with measurable disease in the SOLO3 trial [41], results from ongoing randomised trials are required to reliably assess the contribution of immune checkpoint blockade.

PD-L1 expression and CD8 + T-cell infiltration have previously been associated with better prognosis and/or response to ICIs in ovarian cancer and TNBC [42–44]. Although baseline biomarker data from our study do not allow robust conclusions, the only responding patient with ovarian cancer in Part 1 had the highest PD-L1 expression, and most responding patients with ovarian cancer in Part 2 also had PD-L1 IC ≥ 1%. Furthermore, in Part 2, four of the five patients with a high percentage of CD8 + T cells in the central tumour area achieved either PR or CR. This suggests that pre-existing immunity may enrich response to atezolizumab combination therapy. As expected in ovarian cancer [33], TMB was typically low, and only one patient across all treatment cohorts and arms had TMB ≥ 10 Mut/Mb (accompanied by high CD8 + T cells and high TILs).

The longitudinal biomarker analyses provide a unique opportunity to investigate the tumour intrinsic and microenvironment dynamics in patients receiving a PARPi and ICI in tandem. For tumour intrinsic features, we focused on known PARPi MoAs [1]. We observed decreased DDR and cell cycle and increased apoptosis pathway gene expression—expected MoAs of PARPis— in most responders but not in non-responders post run-in and post-combination. This observation suggests that the efficacy of rucaparib (and potentially combination) treatment is associated with decreased DNA repair and cell proliferation and, consequently, increased cell death. The DDR reduction at initial rucaparib treatment and accumulation of unrepaired DNA might also promote an anti-tumour immune microenvironment [29]. We observed preferential increases in PD-L1 gene and protein expression, CD8 + T-cell activity, and rucaparib-induced cGAS-STING pathway in responders after run-in and/or post-combination. Similar to previous reports that atezolizumab promoted PD-L1 expression in ICs in TNBC [45], we found increased PD-L1 expression at both mRNA and protein levels in ICs, independent of RECIST response status. Despite concerns that PARPi might promote proliferation and stabilise FOXP3 in Tregs, thereby suppressing immunity [46–48], in our study, CD3 + FOXP3+ Treg dynamic changes were not consistently increased upon rucaparib exposure. This suggests that the Treg changes might be related to a feedback of active immunity rather than a PARPi effect, although more in-depth mechanistic studies are required to answer this question definitively.

Exploration of the dynamics of immune modulation by rucaparib alone and with atezolizumab revealed no significantly enriched pathways related to tumour immune microenvironment, probably due to the small sample size and low expression of immune genes. However, consistent with previous findings in ovarian cancer cell lines showing induction of PD-L1 expression by PARPis [30], *CD274* expression increased after rucaparib run-in in three of five responders but not in non-responders. Following the addition of atezolizumab, both responders and non-responders showed higher *CD274* expression, consistent with enhanced PD-L1 expression with atezolizumab [45] independent of response. Increased CD8 + T-cell activity and cGAS-STING pathway activation were seen only in responders after the rucaparib run-in and were sustained or increased further with the addition of atezolizumab in both responders and non-responders.

Since this study was designed, results have been reported from the single-arm open-label Phase II TOPACIO/KEYNOTE-162 study evaluating a different PARPi (niraparib) combined with a different ICI (pembrolizumab) in TNBC or ovarian cancer. Among 55 patients with TNBC (enrolled irrespective of *BRCA* mutation or PD-L1 status), the RECIST ORR was 21% (90% CI: 12–33%), with a higher response rate among patients with *BRCA*_mut_ than *BRCA*_wt_ tumours (47% vs. 11%, respectively; median PFS 8.3 vs. 2.1 months, respectively) [49]. The ORR in 62 patients with platinum-resistant ovarian cancer was 18% (90% CI: 11–29%) and was similar irrespective of *BRCA* mutation status [50]. In the non-randomised Phase Ib/II JAVELIN PARP Medley study evaluating the PARPi talazoparib combined with the PD-L1 inhibitor avelumab, clinical activity was observed primarily in patients with *BRCA*-mutated tumours, with confirmed ORRs of 18% in TNBC and 64% in platinum-sensitive *BRCA-*mutated ovarian cancer [51]. In the Phase IIb tumour-agnostic JAVELIN *BRCA*/*ATM* study evaluating the same combination, durable responses were more common in *BRCA-*associated tumour types (including breast and ovarian cancers) than other tumour types, but the confirmed ORR of 26% was below the pre-specified 40% threshold [52]. Neither of these studies, which enrolled a notable proportion of patients with *BRCA* alterations across a range of tumour types, showed conclusive results on the predictive role of TMB for the PARPi/ICI combination.

An obvious limitation of our study is the small sample size, particularly in the *BRCA*_wt_/LOH_high_ ovarian cancer and TNBC arms, because of premature discontinuation of the study given the changing landscape of ovarian cancer and TNBC, particularly regarding the use of PARP inhibitors in the treatment rather than maintenance setting. Additional limitations include the lack of serial biopsies from a small proportion of patients and reliance on local tumour assessment for molecular characterisation, which in some cases was discordant with central assessment. Only one patient with ovarian cancer in Part 1 and one in Part 2 had a centrally confirmed *BRCA*_wt_/LOH_high_ tumour, precluding conclusions on whether this patient population can benefit from the combination therapy. The lack of reliable information on prior therapy and platinum-free interval in Part 1, which had less strict eligibility criteria, is a weakness and limits context-relevant understanding of the available efficacy information. Another limitation is that the trial was neither designed nor powered to study the effect of the combination on patients with homologous recombination-proficient tumours or to investigate the independent effect of atezolizumab. Furthermore, in the rapidly evolving treatment landscape, most patients with *BRCA-*mutated or HRD tumours will now receive a PARPi as maintenance therapy after front-line therapy, which affects treatment choices and outcomes in the recurrent setting. Thus, the relevance of this combination in recurrent disease is perhaps diminishing. Several Phase III trials are evaluating ICIs and PARPis in various combinations and settings, including ANITA (NCT03598270), ATHENA-COMBO (NCT03522246) [53], DUO-O (NCT03737643), ENGOT ov-44/FIRST (NCT03602859), and ENGOT-ov34/KEYLYNK-001 (NCT03740165), as well as a randomised Phase II trial (NCT02849496) exploring olaparib with or without atezolizumab in *BRCA*-mutated HER2-negative advanced breast cancer. In addition, in the MEDIOLA cohort without germline *BRCA* mutations, a triplet regimen of olaparib, durvalumab, and bevacizumab demonstrated high activity with median OS exceeding 2.5 years [54].

The trial was originally designed to determine the safety and tolerability of the combination and identify a potential recommended dose for further evaluation. Given the changing landscape, the translational research, which was integral to the study from the outset, is now perhaps the most informative aspect of the trial, providing first in vivo evidence of the MoA for this combination. This insight may guide more targeted development of such regimens. Trials randomising patients to PARP inhibition with or without immune checkpoint blockade (for example, the Phase III ATHENA-COMBO trial, which includes a translational research component [53]) will provide further insight into potential synergy. There remain many unanswered questions about the biological interactions of these two classes of agent but the present study represents an important first step. Preliminary signals suggest that activation of the cGAS-STING pathway during treatment may provide an early marker of benefit from therapy. If confirmed, STING pathway activation would provide reassurance that treatment continuation may be beneficial in responding patients, as well as helping to identify patients for whom an early switch to an alternative therapy may be beneficial to avoid unnecessary continuation of ineffective treatment. The identification of more reliable pre-treatment biomarkers to improve patient selection for PARPis and immunotherapy remains an important research goal. In the rapidly evolving treatment landscape for gynaecological cancers, five randomised Phase III trials of anti-PD-L1 agents in newly diagnosed or recurrent ovarian cancer have failed to demonstrate significantly improved outcomes in unselected populations, while offering hints of efficacy in patients with the highest PD-L1 expression [21–25]. Therefore it may be important to limit future trials of immunotherapy agents to populations with high PD-L1 expression, while continuing to explore and develop novel patient selection and therapeutic strategies to improve clinical outcomes.

### Supplementary information

Supplementary material

Protocol

## Data Availability

For eligible studies, qualified researchers may request access to individual patient-level clinical data through a data request platform. At the time of writing, this request platform is Vivli (https://vivli.org/ourmember/roche/). For up-to-date details on Roche’s Global Policy on the Sharing of Clinical Information and how to request access to related clinical study documents, see https://go.roche.com/data_sharing. Anonymised records for individual patients across more than one data source external to Roche cannot, and should not, be linked due to a potential increase in the risk of patient re-identification. All clinical, raw RNA sequencing data are deposited to the European Genome-Phenome Archive under accession number EGAS00001006100.
