# Supplementary material for: PARP inhibition with rucaparib alone followed by combination with atezolizumab: Phase Ib COUPLET clinical study in advanced gynaecological and triple-negative breast cancers
Source: Br J Cancer. 2024 Jul 6;131(5):820–31. doi: 10.1038/s41416-024-02776-7 (PMC11369183; doi:10.1038/s41416-024-02776-7)
Supplement: Supplementary file 1 — Supplementary material [file 41416_2024_2776_MOESM1_ESM.docx]

PARP inhibition with rucaparib alone followed by combination with atezolizumab: phase Ib COUPLET clinical study in advanced gynaecological and triple-negative breast cancers

Rebecca Kristeleit et al.

**Supplementary Materials**

**Supplementary Table 1.** Definition of dose-limiting toxicity: A clinically significant AE (classified according to the NCI CTCAE version 4.03, as applicable) or laboratory abnormality assessed as at least related to the study treatment (rucaparib and/or atezolizumab), unrelated to disease progression, and that meets any of the following criteria:

| ANC <0.5 × 10^9^/L >7 days’ duration or febrile neutropenia (i.e., fever >38.3°C with ANC <1.0 × 10^9^/L) |
| --- |
| Platelet count <25 × 10^9^/L or platelet count <50 × 10^9^/L with bleeding requiring a platelet transfusion |
| Grade 4 anaemia (i.e., life-threatening consequences; urgent intervention indicated) |
| Grade 4 AST, ALT, or total bilirubin that does not resolve to grade ≤2 within 3 weeks of onset and that cannot be attributed to other aetiologies, such as viral hepatitis or disease progression |
| Any non-haematological AE of grade ≥3 except:   - Grade ≥3 skin rash that can be adequately managed with supportive care or resolves to become grade ≤2 within 7 days with appropriate supportive therapy - Alopecia - Grade 3 nausea, vomiting, or diarrhoea if well controlled by systemic medication - Grade 3 immune-mediated AE that resolves to grade ≤1 within 3 weeks of onset - Grade ≥3 fatigue that resolves to grade ≤2 within 7 days - Grade 3 arthralgia that can be adequately managed with supportive care or that resolves to grade ≤2 within 7 days - Grade 3 fever (in the absence of any clinically significant source of fever) that resolves to grade ≤2 within 7 days with supportive care - Grade ≥3 laboratory abnormality that is asymptomatic and deemed by the investigator not to be clinically significant - Grade 3 AST and/or ALT with bilirubin ≤ ULN that resolves to Grade ≤2 within 3 weeks of onset or that can be attributed to other aetiologies, such as viral hepatitis or disease progression - Grade 3 elevation of serum creatinine kinase level that is asymptomatic (i.e., not accompanied by signs, symptoms, or other laboratory abnormalities associated with rhabdomyolysis or myocardial injury) that is deemed by the investigator to be clinically insignificant and that returns to grade ≤2 within 7 days - Grade 3 elevation of serum creatinine that is not accompanied by elevations in BUN or other signs of renal injury or that can be attributed to other aetiologies such as disease-related obstructions - Grade 3 autoimmune thyroiditis or other endocrine abnormality that can be managed by endocrine therapy or hormone replacement - Grade 3 infusion reaction that resolves within 6 hours to grade ≤1 |

*ANC* absolute neutrophil count, *AE* adverse event, *ALT* alanine aminotransferase, *AST* aspartate aminotransferase, *BUN* blood urea nitrogen, *NCI CTCAE* National Cancer Institute Common Terminology Criteria for Adverse Events, *ULN* upper limit of normal.

**Supplementary Table 2.** Summary of baseline and longitudinal tissue clinical biomarker datasets and key biomarker analyses.

| **Biomarker dataset** | **Biomarker analyses** | **Part 1** | **Part 2** | | | |
| --- | --- | --- | --- | --- | --- | --- |
|  |  | **Pre-treatment** | **Pre-treatment** | **Patient-matched pre- vs. post- paired biopsies** | | |
|  |  |  |  | **Run-in vs. pre-treatment** | **Post-combination vs. run-in** | **Post-combination vs. pre-treatment** |
| RNA sequencing | Differential gene expression | NA | BEP = 10 | 9 pairs | 6 pairs | 6 pairs |
|  | IPA pathway analysis |  |  |  |  |  |
| FoundationOne^®^ profiling | *BRCA* mutations, gLOH, TMB, MSI | Retrospective: BEP = 9 | Prospective: BEP = 14 | NA | NA | NA |
| PD-L1 IHC (SP142) | Association with RECIST response and clinical benefit | Retrospective: BEP = 9 | BEP = 10 | 7 pairs | 6 pairs | 5 pairs |
| CD8 IHC | Association with RECIST response and clinical benefit | Retrospective: BEP = 9 | BEP = 10 | 8 pairs | 6 pairs | 6 pairs |
| CD3/FOXP3 duplex immunofluorescence | Effect of rucaparib on Tregs | Retrospective: BEP = 7 | BEP = 10 | 7 pairs | 5 pairs | 5 pairs |

*BEP* biomarker-evaluable population, *gLOH* genomic loss of heterozygosity, *IHC* immunohistochemistry, *IPA* Ingenuity Pathway Analysis, *MSI* microsatellite instability, *NA* not available, *PD-L1* programmed cell death-ligand 1, *RECIST* Response Evaluation Criteria in Solid Tumors, *TMB* tumour mutational burden, *Treg*, T regulatory cell.

**Supplementary Table 3.** Key biological pathway RNA signatures and gene contents**.**

| **Signature** | **Gene content** | **Source and reference** |
| --- | --- | --- |
| DNA damage repair | *DDB2, GTF2H4, GTF2H5, MUTYH, PALB2, RAD54L, RECQL, RNH1, SMUG1, SSBP1, TDP1, TP53BP1, XPC, XRCC2* | Sun *et al.*^1^ |
| Cell cycle | *CCNA1, CCNB1, CCND1, CCND2, CCND3, CCNE1, CCNH, CDC25A, CDK1, CDK2, CDK4, CDK6, CDK7, CDKN1A, CDKN1B, CDKN2A, CDKN2B, CDKN2C, CDKN2D, E2F1, RB1, RBL1, TFDP1* | MSigDb BioCarta_Cell Cycle_Pathway_gene set^2^ |
| Apoptosis | *APAF1, ARHGDIB, BIRC2, BIRC3, CASP1, CASP10, CASP2, CASP3, CASP4, CASP6, CASP7, CASP8, CASP9, DFFA, DFFB, GZMB, LMNA, LMNB1, LMNB2, PARP1, PRF1, XIAP* | MSigDb BioCarta_Caspase_Pathway gene set^2^ |
| CD8 T-cell activity | *GZMA, PRF1* | Rooney *et al.*^3^ |
| cGAS-STING | *CXCL10, CCL5* | Shen *et al.*^4^ |

*cGAS* cyclic GMP–AMP synthase, *STING* stimulator of interferon genes.

**REFERENCES**

1. Sun H., Cao D., Ma X., Yang J., Peng P., Yu M. et al. Identification of a prognostic signature associated with DNA repair genes in ovarian cancer. *Front. Genet.* **10,** 839 (2019).
2. Molecular Signatures Database (MSigDB). BioCarta Cell Cycle Pathway gene set. <https://www.gsea-msigdb.org/gsea/msigdb/cards/BIOCARTA_CELLCYCLE_PATHWAY>
3. Rooney M.S., Shukla S.A., Wu C.J., Getz G., Hacohen N. Molecular and genetic properties of tumors associated with local immune cytolytic activity. *Cell* **160(1-2),** 48–61 (2015).
4. Shen J., Zhao W., Ju Z., Wang L., Peng Y., Labrie M. et al*.* PARPi triggers the STING-dependent immune response and enhances the therapeutic efficacy of immune checkpoint blockade independent of BRCAness. *Cancer Res.* **79,**311–319 (2019).

**Supplementary Table 4.** Treatment exposure.

|  | **Part 1 (dose finding)** | | **Part 2 (dose expansion)** | | | | | |
| --- | --- | --- | --- | --- | --- | --- | --- | --- |
|  | **Cohort 1, 400 mg (*n* = 3)** | **Cohort 2, 600 mg (*n* = 6)** | **Ovarian cancer, Arm A, t*BRCA*_mut_ (*n* = 10)** | | **Ovarian cancer, Arm B, t*BRCA*_wt_/LOH_high_ (*n* = 4)** | | **TNBC, Arm C  (*n* = 5)** | |
|  |  |  | **Run-in** | **Post run-in** | **Run-in** | **Post run-in** | **Run-in** | **Post run-in** |
| **Treatment duration, weeks** | | | | | | | | |
| **Rucaparib** | |  |  |  |  |  |  |  |
| Mean (SD), *n* (%) | 23 (24) | 39 (43) | 4 (3) | 30 (20) | 3 (3) | 19 (11) | 4 (4) | 26 (25) |
| Median (range) | 16 (4–50) | 24 (3–122) | 3 (1.4–11.3) | 24.5 (6–63) | 3 (<1–7) | 18 (7–33) | 3 (<1–11) | 21 (6–69) |
| **Atezolizumab** |  |  |  |  |  |  |  |  |
| Mean (SD) ), *n* (%) | 21 (23) | 36 (43) | – | 27 (20) | – | 10 (9) | – | 25 (25) |
| Median (range) | 13 (4–47) | 23 (3–119) | – | 22 (3–60) | – | 8 (3–21) | – | 18 (6–69) |
| **Dose intensity** | | | | | | | | |
| **Rucaparib** |  |  |  |  |  |  |  |  |
| Mean (SD) ), *n* (%) | 86.6 (27.2) | 60.5 (21.2) | 78.7 (24.1) | 69.2 (24.0) | 68.1 (44.5) | 63.0 (46.6) | 68.8 (41.0) | 58.4 (49.4) |
| Median (range) | 86.4  (59.5–113.8) | 55.0  (39.1–98.0) | 92.1  (40.0–100.0) | 77.3  (14.1–97.6) | 87.2  (2.4–95.5) | 74.8  (2.4–100.0) | 95.2  (2.3–95.5) | 83.0  (2.7–99.7) |
| **Atezolizumab** |  |  |  |  |  |  |  |  |
| Mean (SD) ), *n* (%) | 86.6 (12.1) | 77.6 (14.6) | – | 85.7 (12.5) | – | 64.3 (32.1) | – | 90.9 (6.9) |
| Median (range) | 83.3  (76.5–100.0) | 78.9  (57.1–93.3) | – | 88.8  (66.7­­­–100.0) | – | 75.0  (18.2–88.9) | – | 87.5  (83.3–100.0) |
| **Number of cycles** | | | | | | | | |
| **Rucaparib** |  |  |  |  |  |  |  |  |
| Mean (SD) ), *n* (%) | 8.3 (7.8) | 13.5 (14.2) | – | 10 (7) | – | 7 (3) | – | 9 (8) |
| Median (range) | 6 (2–17) | 8.5 (2–41) | – | 8.5 (2–21) | – | 7 (3–11) | – | 8 (2–23) |
| **Atezolizumab** |  |  |  |  |  |  |  |  |
| Mean (SD) ), *n* (%) | 7.7 (7.4) | 12.8 (14.0) | – | 9.6 (6.6) | – | 4.3 (2.9) | – | 9 (8) |
| Median (range) | 5 (2–16) | 8.5 (2–40) | – | 8 (2–21) | – | 3.5 (2–8) | – | 7 (3–23) |

*LOH* loss of heterozygosity, *mut* mutated, *SD* stable disease, *tBRCA* tumour *BRCA* gene, *TNBC* triple-negative breast cancer, *wt* wild type.

**Supplementary Table 5.** Details of deaths from unknown cause**.**

| **Patient** | **Details** |
| --- | --- |
|  |  |
| 2 | Patient in their 60s died >2 years after last dose of study treatment |
| 12 | Patient in their 40s died >10 months after last dose of study treatment |
| 15 | Patient in their 80s died during carboplatin treatment >3 months after discontinuing study treatment because of clinical progression |
| 22 | Patient in their 40s died >3 months after last dose of study treatment |

**Supplementary Fig. 1 Treg changes by CD3/FOXP3 duplex immunofluorescence in longitudinal biopsies.** Tregs in longitudinal biopsies measured by CD3/FOXP3 duplex immunofluorescence digital counts (left) and by *FOXP3* gene expression (right).

*CR* complete response, *PD* progressive disease, *PFS* progression-free survival, *PR* partial response, *SD* stable disease, *Treg* T regulatory cell.

**Supplementary Fig. 2 Dynamic changes of PD-L1 ICs, tumour-infiltrating CD8+ T cells and Tregs in individual patients.**

*CR* complete response, *IC* immune cell, *PD-L1* programmed cell death-ligand 1, *PR* partial response, *SD* stable disease, *Treg* T regulatory cell.

**
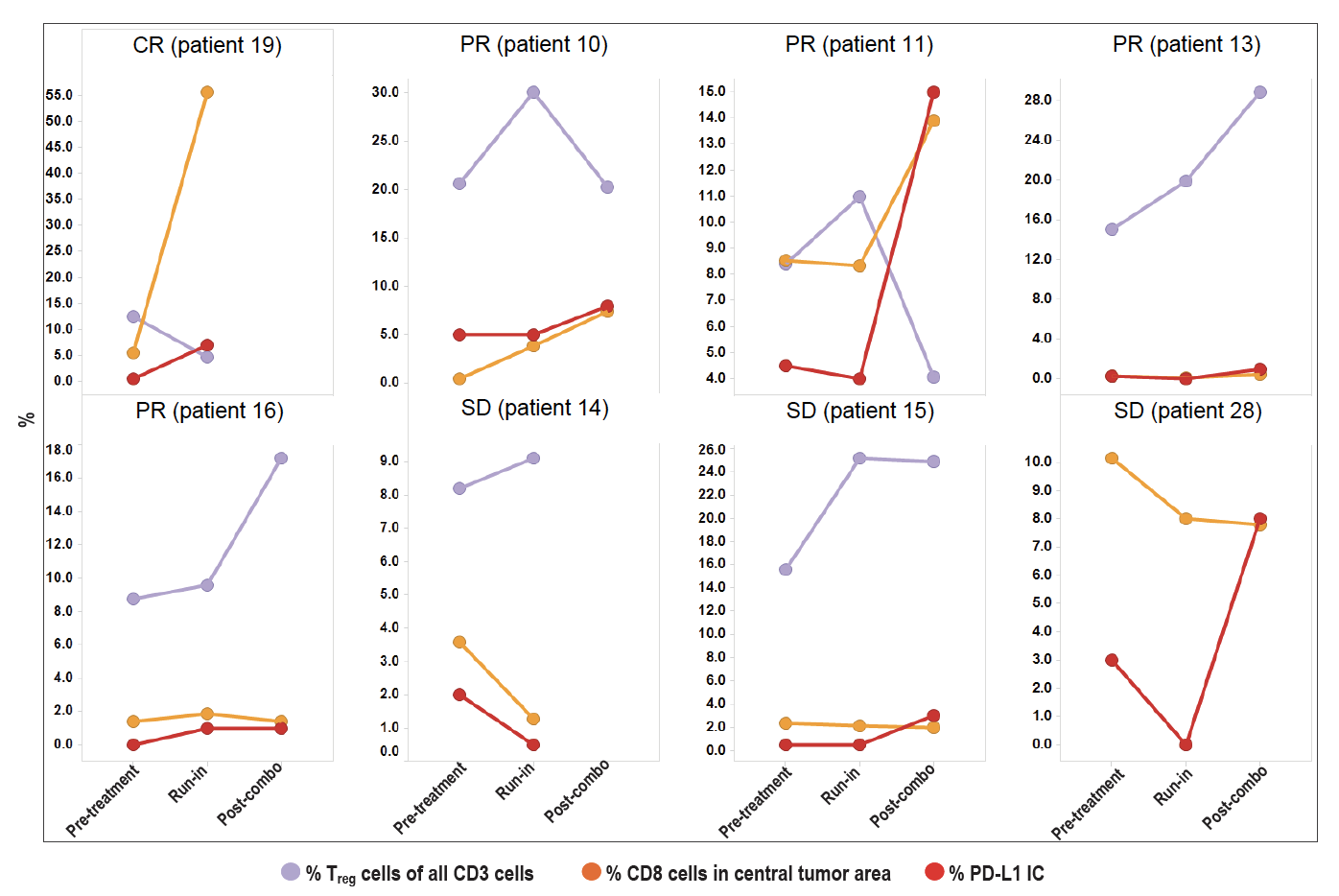
**
