# Supplementary material for: PARP inhibition with rucaparib alone followed by combination with atezolizumab: Phase Ib COUPLET clinical study in advanced gynaecological and triple-negative breast cancers
Source: Br J Cancer. 2024 Jul 6;131(5):820–31. doi: 10.1038/s41416-024-02776-7 (PMC11369183; doi:10.1038/s41416-024-02776-7)

PLEASE READ CAREFULLY:

By opening the attached document you agree to the following terms of use:

- You may not use this document or the information contained herein to a regulatory authority in connection with an application for a marketing authorization or any other regulatory submission without the express written consent of Roche. Please contact global.data_sharing@roche.com

- You may not use this document or the information contained herein to identify clinical trial patients.

- You may not copy, reproduce, or make this document available in any manner that would permit a third-party to review or use the document without first agreeing to these terms of use.

To open the document, please follow the instructions below:

- Please use Adobe Acrobat software to view the document.

- On the left hand panel, open the Attachments tab (paper clip) to see the document attached *(see screenshot below for an example).*

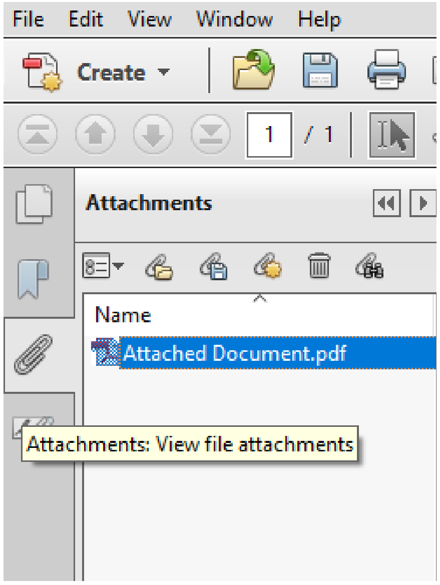

Supplement: Supplementary file 2 — Protocol [file 41416_2024_2776_MOESM2_ESM.pdf]
